# How safe are opioids in the early phase of acute pancreatitis?

**DOI:** 10.1002/ueg2.12545

**Published:** 2024-02-09

**Authors:** Naomi D. E. Thierens, Ihsan Ekin Demir

**Affiliations:** ^1^ Department of Gastroenterology and Hepatology Radboudumc Nijmegen Netherlands; ^2^ Department of Research and Development St. Antonius Ziekenhuis Nieuwegein Netherlands; ^3^ Department of Surgery Klinikum rechts der Isar Technische Universität München Munich Germany

**Keywords:** acute pancreatitis, adverse effects, analgesic therapy, chronic pancreatitis, metamizole, opioids

In the management of acute pancreatitis (AP), adequate pain management is crucial. Nonetheless, literature addressing this aspect of AP is limited. As a result, current guidelines on the management of AP provide only a few recommendations on this topic.^1^ In clinical practice, pain management for AP mimics the approach used for other painful conditions and local protocols. Opioids are often used as they are effective, fast‐acting and generally accepted for the management of acute pain. The article by Pandanaboyana et al.^2^ published in this issue of the UEG Journal raises the question whether opioids are safe in AP patients, as earlier pre‐clinical studies suggest that opioids may suppress the immune response, increase intestinal permeability and cause sphincter of Oddi dysfunction.^3^ They found in a large cohort that AP patients who received opioids during admission were at an increased risk to develop (moderately) severe AP. As this is the largest clinical study being able to demonstrate the association between opioid use and worse outcome of AP, the results are interesting and provoke contemplation. Nevertheless, owing to the observational nature of this study, assessing causality between opioid use and worse outcome of AP is extremely challenging, as more extensive inflammation might result in increased pain severity and therefore necessitate more potent analgesics.

When interpreting the results, it is essential to consider the hypothetical pathophysiological mechanisms underlying worse outcome of AP after opioid use. In early phase AP — up to 2 weeks after disease onset — systemic inflammatory response syndrome (SIRS) is believed to be the primary factor causing (multisystem) organ failure and death.^4^ Based on the occurrence of SIRS, an ongoing randomized controlled trial, aiming to reduce organ failure and death in AP patients, targets the exaggerated immune response using omega‐3 fatty acids (PLANCTON trial EU CT 2022‐000474‐26). Given this context, it is questionable whether suppression of the immune response by admission of opioids in early phase AP should be considered a contributor to the development of severe AP. Moreover, opioids are believed to increase the permeability of the intestine, hypothetically facilitating intestinal bacteria to infect surrounding tissue. In the current study, the authors determined AP severity using the Revised Atlanta Criteria,^5^ which focuses on the occurrence of organ failure and necrosis without differentiating between sterile and infected necrosis. To support this hypothesis, it would be interesting to address the association between opioid consumption and (suspected) infected necrosis rather than the association with necrosis itself.

Over the last decade, the awareness of the adverse effects of opioids has been continually increasing. Previous studies comparing the efficacy and safety of non‐opioids (e.g., metamizole, NSAIDs) versus opioids in AP generally show a slight preference toward opioids, requiring less “escape” medication and no differences in adverse events.^6^, ^7^, ^8^, ^9^ It is essential to consider that these RCTs recruited a limited number of patients, primarily aiming to study pain relief. They are therefore underpowered and lack follow‐up time to evaluate the association between the analgesics studied and the outcome of AP. Overall, a notable number of AP patients will receive sufficient pain relief solely by the use of non‐opioids. Therefore, opioids should be reserved for patients failing on non‐opioids.

In chronic pancreatitis (CP) patients, reluctance to prescribe opioids is more incorporated into clinical practice, which is primarily due to side effects such as dependence and opioid‐induced hyperalgesia.^10^, ^11^ To effectuate sufficient pain relief in CP patients is challenging as available analgesics are limited. Due to its relatively high potency as a level‐one analgesic and favorable gastrointestinal and cardiovascular side‐effect profile compared to classic NSAIDs,^12^, ^13^ metamizole can potentially be a valuable addition to the existing analgesics in this population. Unfortunately, the prescription of metamizole is restricted in several countries due to the extremely rare but potentially severe side effect agranulocytosis.^12^ Epidemiological data indicates that prescription rates of oxycodone are substantially lower in countries where metamizole is widely utilized.^14^ To study the efficacy of metamizole in CP patients and remove legal barriers, the Dutch Pancreatitis Study Group is conducting a double‐blind, placebo‐controlled RCT (MISSION trial EU CT 2023‐504143‐14‐00).

Awareness of the adverse effect of opioids is crucial in both acute and CP. Clinical trials should clarify the implications of opioids in pancreatitis patients and investigate alternative analgesic modalities.

## Data Availability

Data sharing not applicable to this article as no datasets were generated or analyzed during the current study.
